# Design, One Pot Synthesis and Molecular Docking Studies of Substituted-1H-Pyrido[2,1-b] Quinazolines as Apoptosis-Inducing Anticancer Agents

**DOI:** 10.31557/APJCP.2020.21.2.411

**Published:** 2020

**Authors:** Raju Bathula, Shobha Rani Satla, Ramadevi Kyatham, Kiran Gangarapu

**Affiliations:** 1 *Centre for Pharmaceutical Sciences, Institute of Science and Technology, JNTUH, Kukatpally, Hyderabad, *; 2 *Department of Pharmacy, Anurag Group of Institutions, Venkatapur, Ghatkaser, Medchal, Hyderabad, Telangana, India. *

**Keywords:** One Pot, quinazoline, molecular docking, cytotoxicity, anticancer

## Abstract

**Objective:**

The present study focused to build pyridine and quinazoline rings in a single molecule and designed a new fused Pyrido[2,1-b] quinazoline to have a better pharmacological activity.

**Material and Methods:**

A three component, one-pot synthesis of substituted-1H-Pyrido[2,1-b] quinazoline derivatives has been described by conventional and microwave synthesis using triflic acid as catalyst. These compounds were screened for *in vitro* cytotoxic activity against the panel of cancer cell lines A549, NCI-H460, HT-29, HCT-15, DU-145, and HFL.

**Results:**

Among the tested compounds, 11-(1-benzyl-1H-indol-3-y1)-2, 3, 4, 11-tetrahydro-1H-pyrido[2,1-b] quinazoline (**4i**) showed most potent cytotoxicity against A549 and NCI-H460 lung cancer cell lines with IC_50 _values 4.57±0.25 and 5.53±0.49 µM, respectively. Moreover, compound 4i was found to be most potent considerable cell growth inhibition with GI_50_ values of 2.70±0.18 and 3.24±0.40 µM against A549 and NCI-H460 cell lines, respectively. In addition, induction of apoptosis for compound 4i on A549 was investigated by morphological changes, Acridine orange/ethidium bromide (AO/EB) and DAPI staining. Furthermore, a strong anti-clonogenic effect of compound 4i on lung cancer cells was observed. The flow cytometric analysis investigation reveals that compound **4i** arrests the A549 cancer cell lines at the G0/G1 phase of the cell cycle. Molecular docking were also performed on **4i, 4j**, and erlotinib to predict the binding mode towards the EGFR kinase (PDB code: 1M17) and the compounds have displayed similar interactions and compared with erlotinib.

**Conclusion:**

Overall, these findings could suggest that the compound 4i would be an ideal lead as an anticancer agent.

## Introduction

**Figure 1:**
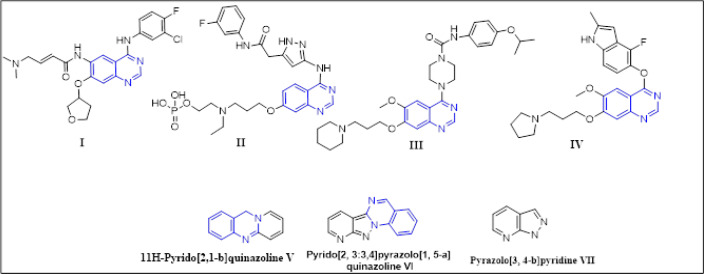
Anticancer Drugs Containing Quinazoline as Core Structural Unit

Cancer is one of the leading causes of death worldwide and accounts for almost 13% deaths than any other infectious diseases (Thun et al., 2010). According to World Health Organization (WHO), projections of cancer prevalence is expected to raise by 21.7 million cases of oncological patients and 13 million deaths by 2030 (El-Azab et al., 2017; Boussari et al., 2018). With the increase in prevalence of cancer and thereby rapidly escalating costs, there are still types of cancer with massive unmet medical needs (Kummar and Takimoto, 2018). Therefore, the development of novel chemotherapeutic agents to fight against this deadly disease is needed urgently (Chakraborty and Rahman, 2012). Nitrogen containing heterocyclic compounds like quinolines and pyridines core rings plays a very important role in drug discovery and development on cancer. (Taylor et al., 2016; Jabir et al., 2018) Some of the marketed drugs have core structure of quinazoline which includes Afatinib (I, as metastatic non-small cell lung cancer) (Shagufta and Ahmad, 2017), Barasertib (II, acute myeloid leukemia)(Helfrich et al., 2016), Tandutinib (III, Antisolid tumors) (Motyckova and Stone, 2015) and Cediranib (IV, Hematological cancers). (Fiedler et al., 2010) The structural moieties of quinazoline exhibit broad range of biological activities viz., anticancer (Zaki et al., 2018), analgesic (Samiksha and Gupta, 2018), antimalarial(Gupta et al., 2018), anti-inflammatory (Moussa et al., 2018) and anticonvulsant activities. (Oluwaseye et al., 2018) Moreover, Pyridine derivatives have known as antifungal, antiviral, anticancer, antidepressant and anti-inflammatory properties (Kurumurthy et al., 2014; Helal et al., 2015) (Figure 1). 

Pyrido [2,1-b]quinazoline (V) is a nitrogen containing fused heterocyclic compound and widely distributed in many bioactive compounds, natural products with interesting biological activities (Tilley et al., 1987; Mikhalev et al., 1995; Gálvez et al., 2018; Samiksha and Gupta, 2018). Pyrido [2,1-b] quinazolines have been discovered for their anticancer, antidepressant, anti-inflammatory, analgesic, antiulcer activities. Recent years fused heterocyclic compounds have gained a renewed attention because of their significant biological activities, some of them Pyrido [2,1-b]quinazoline carboxamide motifs as antiplatelet activity, pyrido [2’,3’:3,4] pyrazolo [1, 5-a] quinazoline (VI) as anticancer and antimicrobial activity (Kumar et al., 2018b) have been reported in the literature. Pyrazolo[3, 4-b]pyridine derivative (VII) have known to possess antitumor, antiviral, antimicrobial and anti-inflammatory activities (Nagender et al., 2014; Zhao et al., 2016) (Figure 1). Previously, many reports have known for versatile one pot, three component reaction of aminopyrimidine, ketone and aldehydes leading to synthesis of diverse fused heterocyclic rings such as Pyrido [2,1-b]quinazoline by using different catalyst (Yang et al., 2013; Sagir et al., 2016). Thus, the present study focused to build pyridine and quinazoline rings in a single molecule and designed a new fused Pyrido [2,1-b] quinazoline to have a better pharmacological activity. The resulting moieties were screened for *in vitro *cytotoxicity by MTT assay and the potent analogs were evaluated for cell growth inhibition assay, Flow cytometric analysis, clonogenic growth inhibition assay, Annexin-V assay for apoptosis.

## Results

*Chemistry*

**Scheme 1:**
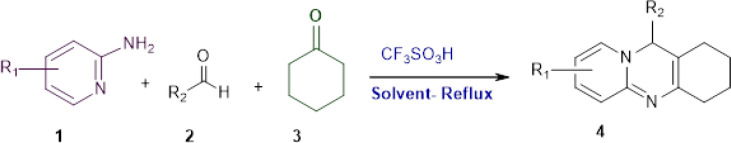
Synthesis of Substituted-1H-Pyrido [2,1-b]Quinazoline

A three component, one pot synthesis of Substituted-1H-Pyrido[2,1-b] Quinazoline derivatives by condensation of 2-aminopyrimidines, substituted aromatic aldehydes and ketones in the presence of ethanol by conventional reflux or by microwave synthesis using triflic acid as catalyst were described in Scheme 1. All the synthesized compounds were purified by column chromatography and characterized by ^1^H,^13^C NMR and Mass spectral data. The physical data of all the synthesized compounds were shown in Table S1 and were compared and authenticated with the previously reported literature. 

**Figure 2:**
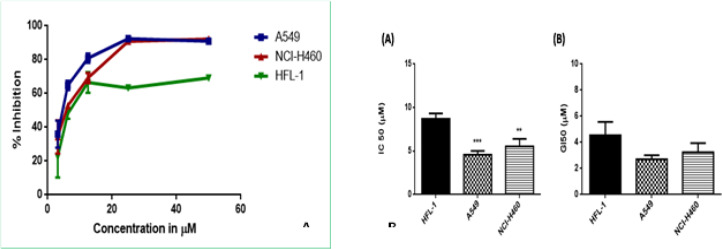
A) Cytospecificity of compound 4i towards lung cancer (A549, NCI-H460) cells compared to normal fibroblasts HFL-1; B) Cytotoxicity and growth inhibitory potential of compound 4i towards lung cancer cells compared to normal fibroblasts HFL-1. Data was expressed as mean ± S.E.M. (n=3). ***p<0.001, **p<0.01, versus normal HFL-1 cell line

To assess the cytotoxic activity of the synthesized compounds in human cancer cells, A549, NCI-H460, HT-29, HCT-15, DU-145 cancer cell lines and HFL-1 normal lung fibroblasts cell lines were employed by using 3-(4,5-dimethylthiazol-2-yl)-2,5-diphenyltetrazolium bromide (MTT) assay. The *in vitro *cytotoxicity studies disclosed that the synthesized compounds manifested assorted anticancer properties referred in Table 1. From the closer analysis of the IC_50_ values, it was observed that compound 4i was more prominent in inducing cytotoxicity in all cancer cells and found to be most potent in A549 lung cancer cell line with IC_50_ value 4.57±0.25 µM whereas, in NCI-H460 cell line 50% inhibition was observed at 5.53±0.49 µM, which indicates that compound has specificity towards lung cancer cell lines, thus we compared the cytotoxicity in normal lung fibroblast cell line HFL-1 where it showed IC_50_ value 8.70±0.36 µM respectively (Figure 2). Hence, the compound was found to have approximately 2-fold selectivity towards the cancer cells. While the compound 4j also exhibited remarkable cytotoxicity with IC_50_ value 7.25±0.38 µM against the colon cancer cell line HT-29, Among the series the compound 4f also showed considerable cytotoxicity and selectivity towards lung cancer with IC_50 _12.84±1.50 and 12.64±0.35 µM in A549 and NCI-H460 cancer cells. These results of biological screening could give a positive hope in anticancer studies that may lead to the origination of novel cytotoxic agents with better activity.

*In vitro growth inhibitory activity*

The synthesized derivatives were further examined for their growth inhibition on potential in human cancer cell lines and normal cell line by MTT assay. The GI_50 _(µM) values (concentration required to inhibit 50% growth of cancer cells) of the tested compounds were listed in Table 2. It is seen from the results that some of the compounds have shown moderate to potent growth inhibition against the tested cancer cell lines. Among this series, from GI_50_ analysis, the compound 4i was found to be the most potent with considerable cell growth inhibition with GI_50_ values of 2.70±0.18 and 3.24±0.40 µM in A549 and NCI-H460 cell lines with selectivity towards lung cancer cells consistent with 50% inhibitory potential, and also have selectivity towards colon cancer cells with significant GI_50 _5.98±0.18, 6.27±0.19µM in HT-29 and HCT-15 cells (Figure 2). Furthermore, the other compounds examined among the series for potential cell growth inhibition, the compounds 4f, 4g, and 4j exhibited promising growth inhibition. The rest compounds were found to be moderately active in inhibiting growth of cancer cells. Hence the compound 4i was further selected for mechanistic studies to unveil the mechanism of cell growth inhibition at the molecular level in A549 cell line. These encouraging results may offer promising support in further mechanistic studies.

*Morphological observations using phase contrast microscope*

**Figure 3:**
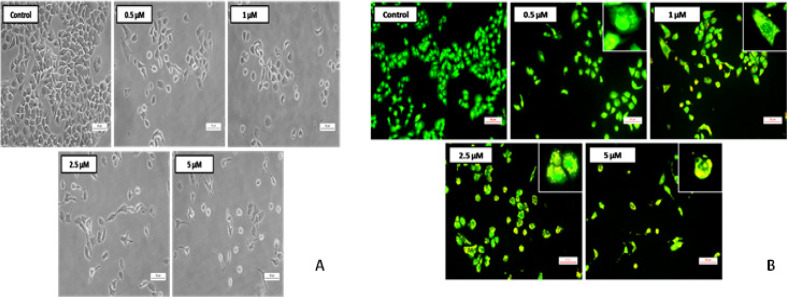
A) Morphological changes observed in Lung cancer cells with compound 4i treatment. A549 cells were treated with and without compound 4i at concentrations 0.5,1, 2.5 and 5 µM. After 72 h the images were captured with a phase contrast microscope at 200X magnification; B) Apoptotic morphology detection by acridine orange-ethidium bromide (AO/EB) fluorescent staining of lung cancer cells after AO and EB staining. Untreated A549 cells showed normal structure without prominent apoptosis and necrosis, cells treated with compound 4i at various concentrations 0.5, 1, 2.5 and 5 µM for 72 h showed prominent apoptotic features like membrane blebbing, nuclear condensation, fragmented nuclei. The images were captured with fluorescence microscope at 200X

The induction of apoptosis and apoptotic cell residues formation is one of the imperative goals in anti-cancer therapeutics (Ward et al., 2008). To examine whether the treatment with the most active compound 4i could lead to the apoptosis induction, A549 cells were treated with the compound 4i, after 72 h of incubation images were captured in phase contrast microscope which delineate the shrinkage of cells and resulted in increased number of dead cells with increase in concentration, whereas these characteristic morphological features were absent in control cells and exhibited healthy morphology as shown in Figure 3.

*Acridine orange/ethidium bromide (AO/EB) staining*

Acridine orange/ethidium bromide (AO/EB) staining assay was performed to differentiate among live, apoptotic and necrotic cells. AO can penetrate the intact cell membrane and stain the nuclei green, whereas EB can only stain the nuclei of cells that have lost membrane integrity and stain orange. Necrotic cells display red fluorescence with no indication of chromatin fragmentation, and the cells also appear swollen (Riyasdeen et al., 2014). It can be inferred from Figure-3B that the control cells exhibited normal cell morphology and appeared green. Whereas, the compound 4i treatment dose dependently clearly induced the apoptotic morphological changes such as cell shrinkage, chromatin condensation, membrane blebbing and apoptotic body formation, indicating that the compound 4i had induced apoptosis in A549 lung cancer cells.

*DAPI staining*

Apoptosis can be demarcated from necrosis by their pronounced change in nuclear appearance. With DAPI staining the untreated cells appeared normally with intact nucleus.

**Figure 4:**
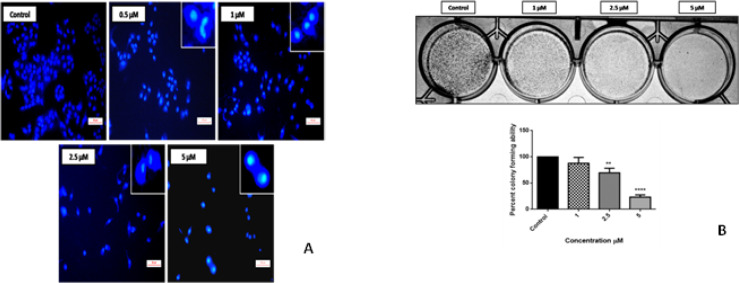
A) Nuclear changes in lung cancer cells after DAPI staining. A549 cells were treated with and without compound 4i at various concentrations 0.5, 1, 2.5 and 5 µM for 72 h then stained with DAPI and compared with control. The treatment showed intact nucleus in untreated cells whereas, altered nuclear architecture with clear apoptotic changes such as bright stain, condensed nuclei were captured with fluorescence microscope at 200X; B) Effect of compound 4i on survival of A549 cell colonies in colony formation assay. A549 cells were treated with compound 4i at 1, 2.5 and 5 µM. After 7 days the colonies were fixed in crystal violet stain, and then cells were counted in Vilber fusion chemdoc imaging system. The picture clearly depicts the strong anti-clonogenic effect of compound 4q on lung cancer cells. The graphical representation illustrates the percent colony forming ability

A549 cells treatment with the compound 4i showed chromatin condensation, nuclear fragmentation, and horse shoe shaped nucleus and bright chromatin due to which are clear morphological indicators of apoptosis as shown in the Figure 4A.

*Effect of compound 4q on the formation of colonies by Clonogenic assay*

The assay mainly tests ability to undergo “unlimited” division by every cell in the population and the hindrance of colony formation by compound 4i was determined in exponentially growing A549 cells by culturing 100-150 cells per well. Treatment with compound 4i was given at different concentrations (1, 2.5 and 5µM), then colonies formation was observed in 7 days’ time period. Then, formed colonies were fixed with glutaraldehyde (6.0% v/v), stained with crystal violet (0.5% w/v). The treatment clearly retained the capacity to produce colonies in lung cancer cell population in a concentration dependent manner. Reductions in the number of colonies were observed as shown in Figure 4B. Hence, these results denote the importance of compound 4i in inhibiting the potential of colony formation. The total colonies were counted by molecular imaging system Vilber Fusion Fx software and the values were represented as a percent colony forming ability.

*Flow-cytometry analysis*

*Effect of compound 4i on mitochondrial membrane depolarization*

**Figure 5:**
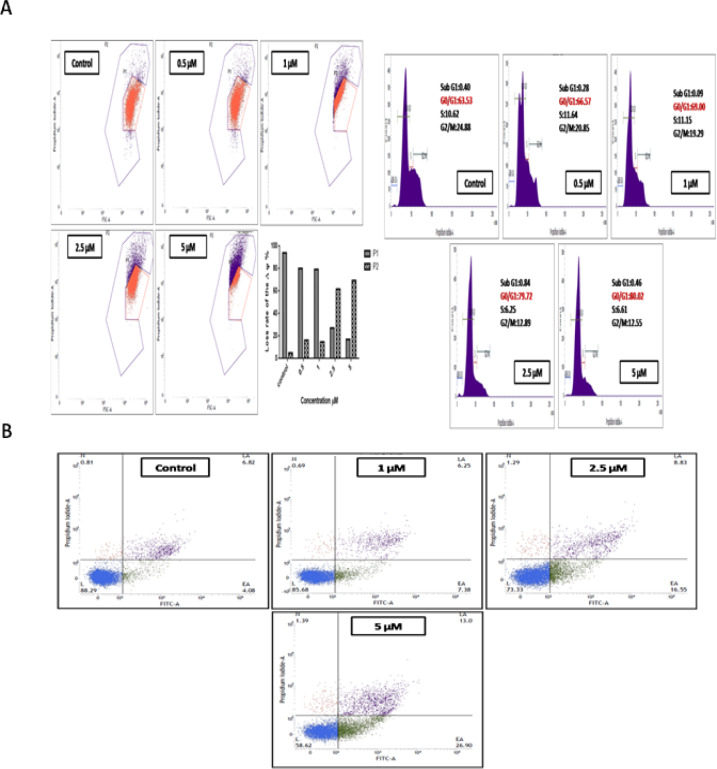
A) Compound 4i reduced ΔΨm in lung cancer cells. A549 cells were treated with various concentrations of compound 4i for 72 h. The control represents the cells without compound 4i treatment. P1 indicates formation of J-aggregates in healthy mitochondria whereas, P2 represents depolarized mitochondria in cells due to the presence of J-monomers; Cell cycle analysis of lung cancer cells following Compound 4i treatment. A549 cells were grown and treated with compound 4i at different concentrations 0.5, 1, 2.5 and 5 µM for 72 h. The cells were harvested, stained with propidium iodide and DNA content was quantified by flow cytometry. Histogram showing the percentage of cells in the Sub G1, G0/G1, G1, S and G2/M phase of the cell cycle obtained after FACS analysis. 10,000 cells were acquired for each sample. B) Apoptosis induction by Compound 4i in lung cancer cells using annexin V FITC/PI staining. A549 cells were cultured and treated with compound 4i ranging from 0.5 to 5µM concentration and incubated for 72 h and processed for annexin V-FITC/ PI double-staining. Quantification of cells undergoing apoptosis or necrosis was carried out with 10,000 cells from each sample and were analyzed by flowcytometry. The percentage of cells positive for Annexin V-FITC and/or Propidium iodide is represented inside the quadrants. Cells in the upper left quadrant (Q1-UL; AV-/PI+): necrotic cells; lower left quadrant (Q2-LL; AV-/PI-): live cells; lower right quadrant (Q3-LR; AV+/PI-): early apoptotic cells and upper right quadrant (Q4- UR; AV+/PI +): late apoptotic cells

The fluorescent orange-red staining is indicative of the presence of polarized mitochondria. On depolarization, there is usually a reduction in orange-red staining occurs. The mitochondria play a fundamental role in initiating the intrinsic pathway of apoptosis in response to many triggers, as it is the leading target of cellular oxidative stress, which impede the electron transport chain, which generate reactive oxygen species (ROS) (Bustamante et al., 2004). Therefore, to test the impact of compound 4i on mitochondria, the ΔΨ_m_ was measured. A549 cells treatment with compound 4i caused significant collapse in the ΔΨ_m_ compared to control cells as shown in the Figure 5A. The compound 4i prompted the depolarisation of ΔΨ_m_ dose-dependently leading to the disruption of electron transport.

*Effect of compound 4q on Cell cycle distribution*

From the *in vitro *screening results, it was evident that the compound 4i showed remarkable toxicity against lung cancer cell line respectively. Hence, in order to reveal whether this cytotoxicity may be due to the phase arrest, cell cycle determination was performed which was parallel with increased percentages of cell death (Żuryń et al., 2016). Cells were treated with compound 4i at concentrations ranging from 0.5 to 5 µM for 48 h, and then the cells were stained with propidium iodide and analyzed by using flow analyzer. The results from Figure 5A indicated that the A549 untreated control cells showed 63.53% cells in G0/G1 phase, whereas compound 4i treatment resulted in significant elevation in G0/G1 population from 63.53% to 80.02% which gradually increased with increase in doses, which implies G0/G1 arrest of the cell cycle The G0/G1 phase arrest was more prominent at 2.5 and 5 µM concentrations.

*Annexin V Dead cell apoptosis assay*

To determine whether the cytotoxicity by compound 4i treatment could induce early apoptotic or late apoptotic cell death, Annexin V-FITC/PI flow cytometry was performed. As appeared in Figure-5B, compound 4i fundamentally exhibited significant early apoptosis, the population of early apoptotic cells increased from 4.08 % (control) to 26.90% concentration. While at 2.5 and 5 μM concentration evidenced both early and late apoptosis.

*Molecular Docking*

The docking of compounds 4i and 4j on EGFR kinase was investigated into the putative binding site of EGFR kinase. Both analogs 4i and 4j were docked using MOE software into the active site of EGFR kinase along with the crystal ligand erlotinib (PDB code: 1M17). All docking calculations were performed and interactions were predicted. The interaction energies of compounds 4i, 4j and erlotinib, docked into the active site of EGFR, were –17.47, -16.57 and -22.34 kcal/mol, respectively Table S2; 

The docking study of the most active compound 4i revealed that the indole ring typically overlaid the corresponding ring of erlotinib without clashing with the surrounding amino acids. The 6-ring and 5-ring of indole nucleus of compound 4i have bound with Val702 with pi-H interaction with distances 3.71 and 4.28 respectively with energies -1.0 and -0.8 kcal/mol respectively. The compound 4i also shows hydrophobic interactions with Phe 699, Leu 694, Met 742 and Leu 764. 

**Figure 6:**
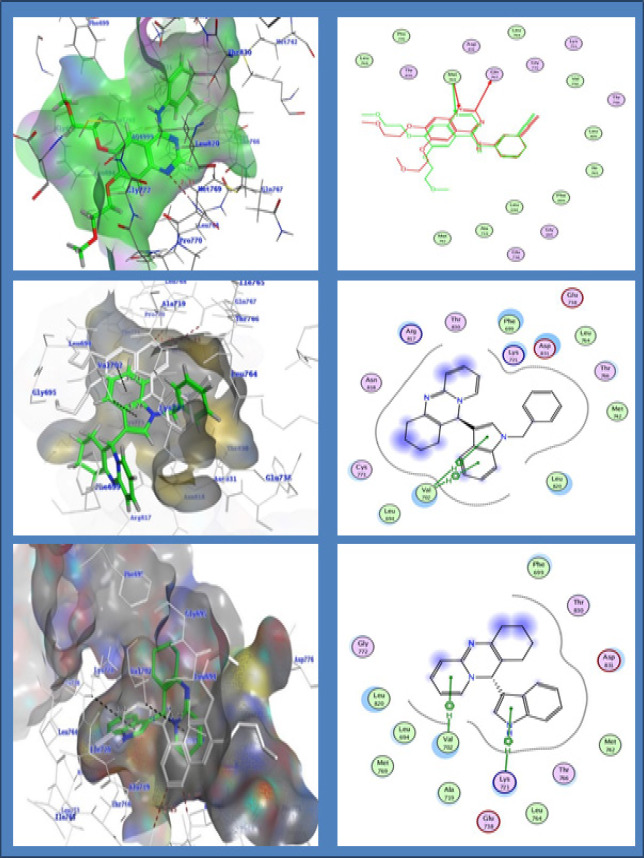
3D and 2D Interactions of Erlotinib with the EGFR Kinase and Overlay Complex with Crystal Ligand; 3D and 2D interactions of compound 4i with the EGFR kinase with pi-H interactions with Val 702: 3D and 2D interactions of compound 4j with the EGFR kinase with pi-H interactions with Val 702 and Lys 721

The indole ring is the main moiety affecting the binding mode of compound 4i in both activation and catalytic loop Figure 6. 

In contrast, compound 4j was bound in similar manner, where the pi-H interaction is observed with quinoline nucleus with Val 702 with -0.6 kcal/mol. Pi-H interactions with Lys 721 indole nucleus with -0.7 kcal/mol. The compound 4j have shown similar hydrophobic interactions with Phe 699, Leu 694, Met 742 and Leu 764. The dock score for 4i, 4j and erlotinib by interactions with EGFR kinase was found to be -6.34, -5.96 and -7.29 respectively. It is clear that the results of the molecular docking can be used to design novel quinoline derivatives with potential anticancer activity and binding to EGFR target. 

*ADME predictions*

The major parameters for pharmacokinetics are absorption, distribution, metabolism and excretion. The in silico ADME properties of substituted 1H-pyrido[2,1-b]quinazoline (4) compounds have shown satisfactory results. Among the evaluated 18 compounds have showed good intestinal absorption. Almost all compounds have shown moderate permeability for *in vitro *Caco-2 cells except 4c and 4i have shown low permeability and low to moderate permeability for *in vitro *MDCK cells except 4h showed 220.117 with high permeability. Predicted In vivo blood-brain barrier penetration demonstrated that compound 4j has high penetration in to CNS and 4c, 4d, and 4h have less penetration ability into CNS. All compounds have strong plasma protein binding the value more than 90% indicates strongly bound and also showed maximum skin permeability. The in silico predicted ADME properties and their values are shown in the Table 3.

**Table 1:** *In vitro* Inhibitory Concentration (IC_50_ µM) Against Human Cancer Cell Lines

Cell line	A549^a^	NCI-H460^b^	HT-29^c^	HCT-15^d^	DU-145^e^	HFL-1^f^
4a	>30	>30	>30	>30	>30	>30
4b	>30	>30	>30	>30	>30	>30
4c	>30	>30	>30	>30	>30	>30
4d	>30	>30	>30	>30	>30	>30
4e	43.08±2.43	43.18±2.58	39.48±1.40	40.91±0.55	23.25±0.25	84.96±3.80
4f	12.84±1.50	12.64±0.35	15.40±0.11	19.26±1.16	16.08±0.14	15.23±0.30
4g	25.54±3.98	41.51±1.27	41.33±1.43	28.59±1.96	32.86±3.77	63.09±1.79
4h	>30	>30	>30	>30	>30	>30
4i	4.57±0.25	5.53±0.49	11.57±0.04	11.31±0.25	9.15±0.09	8.70±0.36
4j	8.98±0.32	9.27±0.54	7.25±0.38	10.88±0.40	12.57±0.23	9.31±0.32

^a^A549, adenocarcinoma; ^b^lung carcinoma; ^c^colorectal cancer; ^d^colon cancer cell line; ^e^prostate cancer cell line; ^f^non-small cell lung cancer

**Table 2:** *In vitro* Growth Inhibitory Concentration (GI_50_ µM) Against Human Cancer Cell Lines

Cell line	A549^a^	NCI-H460^b^	HT-29^c^	HCT-15^d^	DU-145^e^	HFL-1^f^
4a	>30	>30	>30	>30	>30	>30
4b	>30	>30	>30	>30	>30	>30
4c	>30	>30	>30	>30	>30	>30
4d	>30	>30	>30	>30	>30	>30
4e	29.53±0.79	20.83±1.82	15.75±0.97	23.44±0.20	17.7±0.13	68.64±3.20
4f	8.64±0.40	9.62±0.41	11.43±0.06	12.94±0.73	11.35±0.34	11.63±0.17
4g	9.23±0.93	17.48±2.05	20.81±3.74	10.03±0.97	7.37±2.50	41.69±0.98
4h	>30	>30	>30	>30	>30	>30
4i	2.70±0.18	3.24±0.40	5.98±0.18	6.27±0.19	9.64±0.37	4.54±0.59
4j	5.79±0.14	7.10±0.19	4.40±0.20	7.81±0.65	8.10±0.10	7.67±0.22

**Table 3:** In Silico ADME Properties Predicted for 20 Novel Compounds

Compounds	Human intestinal absorption (%)	in vitro Caco-2 cell permeability (nm/sec)	in vitro MDCK cell permeability (nm/sec)	in vitro Plasma protein binding (%)	in vivo blood-brain barrier penetration (C.brain/C.blood)	Skin Permeability
4a	100.00	47.55	22.36	90.03	1.65	-2.95
4b	100.00	54.46	12.36	99.22	2.73	-3.03
4c	98.45	1.68	7.35	91.26	0.19	-3.26
4d	100.00	57.96	25.05	92.43	0.57	-3.31
4e	100.00	35.84	19.16	89.60	2.28	-2.93
4f	100.00	47.91	8.35	100.00	3.68	-3.07
4g	100.00	55.03	26.92	100.00	2.53	-3.42
4h	97.60	56.83	220.12	88.73	0.10	3.70
4i	100.00	32.34	13.75	100.00	2.17	-2.41
4j	94.39	27.22	17.45	93.52	9.38	-3.59

## Discussion

In conclusion, we developed substituted 1*H-pyrido *[2,1-b]quinazoline (4) derivates from the various 2-aminopyrimidines, substituted aromatic aldehydes and ketones by using recyclable and it under go one pot the component system to formation of Pyrido[2,1-b]quinazoline (4) compounds are more efficient and simple method for the organic chemistry synthesis. The synthesized compounds were screened for *in vitro *cytotoxicity by MTT assay against the panel of human cancer cell lines A549, NCI-H460, HT-29, HCT-15, DU-145 cancer cell lines and HFL-1 normal lung fibroblasts cell line. Among the tested compounds, 11-(1-benzyl-1H-indol-3-y1)-2, 3, 4, 11-tetrahydro-1*H-pyrido* [2,1-b] Quinazoline (4i) showed potent cytotoxicity in A549 and NCI-H460 lung cancer cell lines with IC_50 _values 4.57±0.25 and 5.53±0.49 µM, respectively. The induction of apoptosis was examined by morphological changes of compound 4i in A549 cancer cells and showed microscopically shrinkage of cells with increased number of dead cells in dose dependent manner. AO/EB and DAPI staining studies revealed at compound 4i treatment dose dependently clearly induced the apoptotic morphological changes such as cell shrinkage, chromatin condensation, membrane blebbing and apoptotic body formation, indicating that the compound 4i had induced apoptosis in A549 lung cancer cells. Colony formation in A549 was inhibited by compound 4i in a concentration dependent manner. Furthermore, the flow cytometric investigation using compound 4i in A549 cell line in a dose dependent manner gave the following illustrations, 1) Cancer cells were arrested at the G0/G1 Phase of cell cycle.2) Evidence of the depolarization of ΔΨ_m_ dose-dependently leading to the disruption of electron transport chain. 3)Annexin V-FITC/PI flow cytometry exhibited significant early apoptosis. The molecular docking studies on EGFR kinase results indicated that two compounds 4i and 4j of binding interactions strongly correlated with crystal ligand. Overall, these findings could suggest that these compounds would be an ideal motif’s as an anticancer agent. 

*Acknowledgment*

The authors are thankful to Director, CPS, IST, JNTUH for providing the necessary facilities and also thankful to UGC for financial support. 

*Experimental*

*Chemistry*

All chemicals and reagents were obtained from Aldrich Lancaster (Alfa Aeser, Johnson Matthey Company, Ward Hill, MA, USA), or Spectrochem Pvt. Ltd. (Mumbai, India) and were used without further purification. Reactions were performed by TLC on silica gel glass plate containing 60 GF-254, and visualization was achieved by UV light or iodine indicator. ^1^H and ^13^C NMR spectra were determined in CDCl_3_ by using Varian and Avance instruments of 400Hz. Chemical shifts are expressed in parts per million ( in ppm) downfield from internal TMS and coupling constants are expressed in Hz. ^1^H NMR spectroscopic data coupling constants in Hz, number of protons. ESI mass spectra were recorded on a Micro mass Quattro LC using ESI+ software with capillary voltage 3.98 kV and an ESI mode positive ion trap detector. Melting points were determined with an Electro thermal melting point apparatus.

*General procedure*

*Preparation of Pyrido [2,1-b]quinazoline (4)*

A One-pot and three component reaction were employed for the preparation of final compound (4) briefly, 2-aminopyridine (1, 1.0 mmol), aldehydes (2, 1.0 mmol) and ketones (2.0 mmol) was dissolved in EtOH in presence of CF_3_SO_3_H as a catalyst under nitrogen condition the reaction mixture was stirred with reflux for 5 hours at 70^o^C by conventional method or 35 minutes under microwave irradiation, the completion of reaction was monitored by the TLC. Then solvent was removed from the reaction by rotavaccum pressure. Resultant reaction mixture extracted with ethyl acetate and water, the organic layer washed with sodium sulphate then it is evaporated to obtain solid compound later purified by the column chromatography by using Ethyl acetate and Hexane (80:20) as mobile phase to afford a final desired product. 

*Spectral data*

*11-Phenyl-2, 3, 4, 11-tetrahydro-1H-pyrido [2, 1-b] quinazoline (4a)*

m.p: 154–156 ^o^C; ^1^H NMR: δ 7.36–7.28 (m, 5H), 6.89 (t, 1H, J = 7.8), 6.73–6.69 (m, 2H), 5.98 (t, 1H, J = 6.6), 5.37 (s, 1H), 2.38–2.24 (m, 2H), 1.74–1.59 (m, 6H); ^13^C NMR: δ 148.7, 142.6, 136.8, 135.3, 133.1, 128.7, 128.2, 126.8, 123.6, 108.1, 106.6, 68.3, 30.3, 26.6, 23.1, 22.6; MS: m/z 263.0 [M + H+].

*11-(4-Chlorophenyl)-2, 3, 4, 11-tetrahydro-IH-pyrido [2, 1-b] quinazoline (4b)*

mp: 88–93 ^0^C; ^1^H NMR: δ 7.50–7.21 (m, 7H), 6.62 (t, 1H, J = 6.0), 5.64 (s, 1H), 2.47–2.36 (m, 2H), 1.88–1.61 (m, 6H); ^13^C NMR: δ 148.8, 141.9, 137.5, 135.2, 134.5, 133.0, 129.4, 128.3, 123.6, 108.3, 106.8, 67.8, 29.6, 26.4, 23.0, 22.6; MS: m/z 297.0 [M + H+]. 

*11-(4-Nitrophenyl)-2, 3, 4, 11-tetrahydro-1H-pyrido[2,1-b]quinazoline (4c)*

mp: 150–151 ^o^C; ^1^H NMR: δ 8.25–8.22 (m, 2H), 7.54–7.45 (m, 3H), 7.34–7.29 (m, 1H), 7.07 (d, 1H, J = 6.3), 6.46 (t, 1H, J = 6.3), 5.72 (s, 1H), 2.53–2.37 (m, 2H), 1.91–1.61 (m, 6H); MS: m/z 308.12 [M + H+].

*11-(4-Methoxyphenyl)-2, 3, 4, 11-tetrahydro-1H-pyrido[2,1-b]quinazoline (4d)*

mp: 88–89 ^o^C; ^1^H NMR: δ 7.27–7.21 (m, 2H), 6.88–6.84 (m, 3H), 6.76–6.68 (m, 2H), 5.99 (t, 1H, J = 6.6), 5.32 (s, 1H), 3.79 (s, 3H), 2.39–2.29 (m, 2H), 1.77–1.57 (m, 6H); ^13^C NMR: δ 159.7, 148.9, 136.6, 135.4, 135.1, 133.4, 128.3,123.5, 114.2, 108.6, 107.0, 67.9, 55.3, 30.2, 26.7, 23.2, 22.8; MS: m/z 295.0 [M+ H+].

*(p-Tolyl)-2, 3, 4,11-tetrahydro-1H-pyrido[2,1-b]quinazoline (4e)*

mp: 125–126 ^o^C; ^1^H NMR: δ 7.24–7.11 (m, 4H), 6.87 (t, 1H, J = 7.8), 6.73–6.65 (m, 2H), 5.96 (t, 1H, J = 6.6), 5.32 (s, 1H), 2.32–2.30 (m, 5H), 1.79–1.58 (m, 6H); ^13^C NMR: δ 148.9, 139.8, 138.2, 136.5, 135.4, 133.4, 129.6, 126.9, 123.5, 108.5, 106.9, 66.2, 30.2, 26.7, 23.2, 22.7, 21.2; MS: m/z 276.9 [M + H+].

*8-Chloro-11-(4-chlorophenyl)-2, 3, 4, 11-tetrahydro-1H-pyrido [2, 1-b] quinazoline (4f)*

mp: 131–132 ^o^C; ^1^H NMR: δ 7.35–7.32 (m, 2H), 7.27–7.22 (m, 2H), 6.89–6.85 (m, 1H), 6.78 (s, 1H), 6.75 (d, 1H, J = 2.1), 5.32 (s, 1H), 2.34–2.23 (m, 2H), 1.77–1.59 (m, 6H); ^13^C NMR: δ 146.8, 140.4, 136.6, 134.9,134.8, 132.5, 129.4, 128.3, 124.6, 115.3, 107.4, 68.0, 29.9, 26.6, 22.9, 22.5; MS: m/z 330.8 [M + H+].

*8-chloro-11-(4-fluorophenyl)-2,3,4,11-tetrahydro-1H-pyrido[2,1-b]quinazoline (4g)*

mp: 88–93 ^0^C; ^1^H NMR: δ 7.50–7.21 (m, 7H), 6.62 (t, 1H, J = 6.0), 5.64 (s, 1H), 2.47–2.36 (m, 2H), 1.88–1.61 (m, 6H); ^13^C NMR: δ 148.8, 141.9, 137.5, 135.2, 134.5, 133.0, 129.4, 128.3, 123.6, 108.3, 106.8, 67.8, 29.6, 26.4, 23.0, 22.6; MS: m/z 314.0 [M + H+]. 

*11-(4-trimethoxyphenyl)-2, 3, 4, 11-tetrahydro-1H-pyrido[2,1-b] quinazoline (4h)*

mp: 89–92 ^o^C; ^1^H NMR: δ 7.32–7.28 (m, 2H), 7.10–6.85 (m, 3H), 6.75–6.65 (m, 2H), 6.10 (s, 1H), 5.75 (d, 1H, J = 2.1), 3.85 (s, 3H), 2.34–2.23 (m, 2H), 1.77–1.59 (m, 6H); ^13^C NMR: δ 160.0, 147.2, 135.7, 134.6, 134.1, 132.5, 127.5, 124.5, 115.2, 107.6, 106.8, 66.8, 56., 30.6, 27.0, 24.0, 23.6; MS: m/z 295.5 [M + H+].

*11-(1-benzyl-1H-indol-3-yl)-2, 3, 4, 11-tetrahydro-1H-pyrido [2, 1-b] quinazoline (4i)*

mp: 185–186 ^o^C; ^1^H NMR: δ 8.20- 7.80(m, 3H), 7.50-7.35 (m, 4H), 7.20-7.05 (m, 5H), 6.65 (s, 1H), 6.47 (1H, dd, J = 8.3, 1.9 Hz), 5.99 (1H, d, J = 10.1 Hz), 5.55-5.25 (s, 2H), 2.21-2.94 (m, 3H), 2.00 (m, 1H), δ 1.56-1.85 (m,4H). ^13^C NMR: δ 156.8, 147.8, 137.4, 134.9,134.8, 132.5, 129.4, 128.3, 124.6, 115.3, 107.4, 68.0, 29.9, 26.6, 22.9, 22.5; MS: m/z 392.46 [M + H+].

*11-(1H-indol-3-yl)-2, 3, 4, 11-tetrahydro-1H-pyrido [2,1-b]quinazoline (4j)*

mp: 180–182 ^o^C; ^1^H NMR: δ 7.89–7.32 (m, 4H), 7.27–7.22 (m, 3H), 6.89–6.85 (m, 2H), 6.78 (s, 1H), 6.75 (d, 1H, J = 2.1), 2.34–2.23 (m, 2H), 1.77–1.59 (m, 6H); ^13^C NMR: δ 146.8, 140.4, 136.6, 134.9,134.8, 132.5, 129.4, 128.3, 124.6, 115.3, 107.4, 68.0, 29.9, 26.6, 22.9, 22.5; MS: m/z 301.25 [M + H+].

*Biological Activity*

*Materials and Methods*

*Cell culture*

Various cancer cells were used to screen anticancer activity of newly synthesized compounds. Lung (A549 and NCI-H460), colon (HCT-15 and HT-29), prostate (DU-145) cancer cells maintained in RPMI-1640 media while normal lung fibroblasts cell line (HFL-1) were maintained in F-12 k medium supplemented with 10% fetal bovine serum (FBS) with 1% antibiotic-antimycotic solution (Sigma). Cells were maintained in 5% CO2 with 98% relative humidity at 37oC in incubator. When 80-90% of confluency is reached, they were sub-cultured using 0.25% trypsin/1 mM EDTA solution for further passage. The compounds in this series were dissolved in DMSO (1 %) to prepare the stock solution of 10 mM. Further dilutions were made accordingly with respective media to obtain the required concentrations.

*Cytotoxicity assay*

MTT assay measures the reduction of MTT (3-(4,5-dimethylthiazol- 2-yl)-2,5-diphenyl tetrazolium bromide) by mitochondrial succinate dehydrogenase enzyme to insoluble formazan crystals. Since reduction of MTT occurs only in metabolically active cells, the level of activity is the measure of the viability of the cells.(Pordeli et al., 2017). Briefly, cells were plated in 96-well plates at a density of 3,000 to 6,000 cells per well in 100 µl of complete medium and allowed to grow overnight. Then the cells were treated with different concentrations of the synthesized compounds and incubated for 72 h. After the treatment, 100 µl of MTT (0.5 mg/ml) was added and incubated at 37^o^C for 4 h. Then MTT reagent was aspirated and formazan crystals formed were dissolved by the addition of 200 µL of DMSO for 20 min at 37^o^C. The formazan product quantity was measured by using a spectrophotometric microtiter plate reader (Spectra Max, M4 Molecular devices, USA) at 570 nm wavelength(Nekkanti et al., 2017).

*Cell Growth inhibition assay*

Briefly, the cells were seeded on to a 96-well plate in 100 µl of medium. Following 24 h MTT reagent was added in few wells and day 0 absorbance was analyzed, and then cells were treated with compounds and incubated for an additional 72 h. After the treatment, 100 µl of MTT (0.5 mg/ml) was added and incubated at 37^o^C for 4 h. Then MTT reagent was suctioned, the insoluble formazan crystals were dissolved in DMSO and formazan product formed is measured at 570 nm wavelength. The day 0 absorbance was subtracted from the 72 h incubated plates and data were plotted as a percentage of untreated control (Dai et al., 2011).

*Morphological observation*

A549 cells were plated in 12 well culture plates with a cell density of 1.2x10^5 ^cells/ml and allowed to adhere for 24 h. Then the cells were incubated with 0.5, 1, 2.5 and 5 µM concentrations of compound 4q. After 72 h treatment, cells were monitored for the morphological changes and the images were captured under a phase contrast microscope (Nikon, Inc. Japan) at 200X magnification.

*Acridine orange ethidium bromide (AO/EB) staining*

A549 cells were seeded at a concentration of 1.2x10^5 ^cells/ml and treated with various concentrations of compound 4q and the plates were incubated for 72 h. Then, 100 µl from fluorescent dyes comprising both Acridine Orange (Helfrich et al., 2016) and Ethidium Bromide (Vundru et al., 2013) were added into each well in equal volumes (10 µg/ml) respectively (Nekkanti et al., 2017), then the cells were Immediately visualized under fluorescence microscope with excitation (488 nm) and emission (550 nm) at 200X magnification. 

*4′,6-diamidino-2-phenylindole nucleic acid staining (DAPI)*

Morphological changes in the nucleus by the cytotoxic compound 4q treatment were observed with the DAPI staining as per the method described with small modifications. After treatment with compound 4q for 72 h, lung cancer cell line A549 were washed with PBS then fixed with 4% formaldehyde for 10 min and then permeabilized with 0.1% Tween 20 followed by staining with 1 µM DAPI. Control and treated cells were observed under fluorescence microscope with excitation at 359 nm and emission at 461 nm using DAPI filter at 200X magnification (Sgorbati et al., 1986). 

*Flow cytometric analysis*

*Measurement of mitochondrial membrane potential*

The cyanine dye JC-1 (5,5′,6,6′-tetrachloro-1,1′,3,3′-tetraethylbenzimidazolylcarbocyanine iodide) is a cell-penetrating dye that facilitates discrimination of energized and de energized mitochondria thus, high mitochondrial potential shifts its emission fluorescence from green to red (Vundru et al., 2013).This phenomenon was assessed by flow cytometry (FACS). A549 cells (1.5x10^6 ^cells/ml) were seeded in 12 well plates and allowed to adhere for overnight. Then the cells were incubated with compound 4q at 0.5, 1, 2.5 and 5 µM concentrations for 72 h. Cells were collected and washed with PBS and resuspended in solution of JC- 1 (1 µM) and incubated for 30 min in incubator at 37^o^C. The cells were washed twice with PBS and cells were trypsinized, centrifuged and 10,000 events were analyzed by flow cytometer (BD FACSVerse™, USA).

*Cell cycle analysis*

In order to reveal whether arrest of cell cycle was mechanistically responsible for sensitizing lung cancer cells towards apoptosis, the flow cytometric analysis was performed to analyse the distribution of the cell population in various cell cycle phases (Mani et al., 2017). Here, A549 cancer cells were incubated with compound 4q at various concentrations ranging from 0.5 to 5 µM for 48 h. Untreated and treated cells were harvested, washed and fixed overnight in 70% ethanol in PBS at -20^o^C. Fixed cells were pelleted and stained with cell cycle analysis reagent propidium iodide (50 μg/ml) with RNase A for 20 min at 37^o^C in dark and about 10,000 events were acquired and analyzed on a flow cytometer (Kumar et al., 2018a).

*Annexin assay*

A fundamental part of apoptosis is the flipping of phosphatidyl serine (PS) from the inner surface to the outer surface of the plasma layer of the cells. PS is a component of phospholipid usually located on the cytoplasmic surface of the cell layer in viable cells. When the apoptotic condition is initiated in a cell, PS is no more restricted to the cytosolic region and will flip on the surface of the cell. Translocation of PS is considered to be a hall mark of apoptosis (Zhu et al., 2015). To experimentally address this phenomenon briefly, 1 X 10^5^ cells were seeded in a 12-well plate and treated with different concentration of compound 4q and incubated for 72 h. Then untreated and treated cells were harvested and the cells were processed with annexin V-FITC and Propidium Iodide (PI) staining (Bio Legend), according to the manufacturer’s instructions. Further, early/late apoptosis and necrosis parameters were analyzed with quadrant statistics on propidium iodide-negative cells, fluorescein positive cells and propidium iodide-positive cells, respectively. 

*Clonogenic growth inhibition assay*

Lung cancer cells A549 at exponentially phase were seeded into 12-well culture plates and kept overnight and treated with the compound 4q. Every 2 days the medium was replaced with the fresh medium. After 7 days of incubation, the colonies formed were fixed and stained with 1 % crystal violet in methanol for 3 h. The number of stained colonies were counted under chemdoc imaging system (Vilber Fusion Fx, France). Colony formation was calculated as a percentage to untreated control cultures (Moghaddam et al., 2009).

*Molecular Docking Studies*

In this molecular docking study were carried out to examine the possible interactions with target enzyme using Dock Methodology of computational DOCK software. The docking methodology consists of many parameters such as target selection and preparation, isolation of binding cavity with site finder, preparation of ligands, and finally docking to its receptor. The crystal structure of a-amylase was retrieved from Protein Data Bank (PDB ID: 1M17) having a co-crystal ligand erlotinib and water molecules are removed and protein structure were energy minimized using default settings. The ligands are built using builder in Marvin sketch and energy minimized using MMFF94x force field. The docking protocol was carried out with ligand mbd file, Triangle Matcher as Placement, Rescoring using London dG scoring and finally optimized poses are ranked using GBVI/WSA DG score. The docking poses were browsed visually and best interactions were isolated and computed with ligand interactions (El-Azab et al., 2017).

*ADME Predictions*

The in silico ADME properties of these synthesized compounds were calculated by using the online server preADMET (http://preadmet.bmdrc.org/). The ADMET properties, human intestinal absorption (HIA), Caco-2 cell permeability, Maden Darby Canine Kidney (MDCK) cell permeability, plasma protein binding and blood brain barrier penetration (BBB) were predicted using this program.
